# Serum 25-hydroxyvitamin D threshold and risk of rickets in young children: a systematic review and individual participant data meta-analysis to inform the development of dietary requirements for vitamin D

**DOI:** 10.1007/s00394-023-03299-2

**Published:** 2024-01-27

**Authors:** Magali Rios-Leyvraz, Tom D. Thacher, Aashima Dabas, Heba Hassan Elsedfy, Giampiero I. Baroncelli, Kevin D. Cashman

**Affiliations:** 1 Department of Nutrition and Food Safety, World Health Organization, Geneva, Switzerland; 2 Department of Family Medicine, Mayo Clinic, Rochester, Minnesota USA; 3 Department of Pediatrics, Maulana Azad Medical College, New Delhi, India; 4 Department of Pediatrics, Ain Shams University, Cairo, Egypt; 5 Pediatric and Adolescent Endocrinology, Division of Pediatrics, Department of Obstetrics, Gynecology and Pediatrics, University Hospital, Pisa, Italy; 6 Cork Centre for Vitamin D and Nutrition Research, School of Food and Nutritional Sciences, and Department of Medicine, University College Cork, Cork, Ireland

**Keywords:** Serum 25-hydroxyvitamin D, Children, Rickets, 25OHD, Vitamin D threshold

## Abstract

**Purpose:**

The objective of this systematic review was to determine a minimum serum 25-hydroxyvitamin D (25OHD) threshold based on the risk of having rickets in young children. This work was commissioned by the WHO and FAO within the framework of the update of the vitamin D requirements for children 0–3 years old.

**Methods:**

A systematic search of Embase was conducted to identify studies involving children below  4 years of age with serum 25OHD levels and radiologically confirmed rickets, without any restriction related to the geographical location or language. Study-level and individual participant data (IPD)-level random effects multi-level meta-analyses were conducted. The odds, sensitivity and specificity for rickets at different serum 25OHD thresholds were calculated for all children as well as for children with adequate calcium intakes only.

**Results:**

A total of 120 studies with 5412 participants were included. At the study-level, children with rickets had a mean serum 25OHD of 23 nmol/L (95% CI 19–27). At the IPD level, children with rickets had a median and mean serum 25OHD of 23 and 29 nmol/L, respectively. More than half (55%) of the children with rickets had serum 25OHD below 25 nmol/L, 62% below 30 nmol/L, and 79% below 40 nmol/L. Analysis of odds, sensitivities and specificities for nutritional rickets at different serum 25OHD thresholds suggested a minimal risk threshold of around 28 nmol/L for children with adequate calcium intakes and 40 nmol/L for children with low calcium intakes.

**Conclusion:**

This systematic review and IPD meta-analysis suggests that from a public health perspective and to inform the development of dietary requirements for vitamin D, a minimum serum 25OHD threshold of around 28 nmol/L and above would represent a low risk of nutritional rickets for the majority of children with an adequate calcium intake.

**Supplementary Information:**

The online version contains supplementary material available at 10.1007/s00394-023-03299-2.

## Background

Vitamin D is an essential nutrient for bone health [1] and possibly for other extra-skeletal health outcomes [1, 2]. The main sources of vitamin D are dietary intake and dermal synthesis during sunlight exposure [3]. Vitamin D (along with calcium and zinc) has been prioritized by the Food and Agriculture Organization (FAO) together with the World Health Organization (WHO), as part of the update of their 2004 nutrient requirements for children aged 0–3 years [4, 5]. Dietary Reference Values (DRV) for vitamin D, as estimates of the dietary requirements for the vitamin, are crucial from a public health perspective in providing a framework for the prevention of vitamin D deficiency and optimizing vitamin D status of individuals [6]. With the vitamin D DRV update in mind, a recent FAO-WHO-commissioned systematic review and meta-analysis evaluated circulating 25-hydroxyvitamin D (25OHD), parathyroid hormone and other newer potential biomarkers of vitamin D status (such as free and bioavailable 25OHD, 24,25-dihydroxyvitamin D, C3-epimer of 25OHD, and vitamin D_3_) in terms of their use in defining dietary requirements for vitamin D in young children [7]. The systematic review concluded that circulating 25OHD is a robust and reliable marker of vitamin D status in infants and children [7].

In setting DRVs for vitamin D, there is a need to clarify the relationship of serum 25OHD and the reference level of the critical indicator(s) of health outcomes for nutrient adequacy, taking into consideration sex, life-stage and vulnerable groups [8]. This serum 25OHD threshold, in turn, is used to establish the recommended vitamin D intake which maintains a stated percentage individuals above this threshold, and thus ensuring adequacy. For infants and children, the FAO-WHO prioritized the risk of rickets as the critical indicator amongst other skeletal and extra-skeletal health outcomes [9]. Rickets is a softening and weakening of bones at the growth plate, which can lead to painful and long-term health consequences [10], including potentially life-threatening complications [11]. It can be diagnosed based on clinical signs, biochemical tests and radiographies [10]. Several authorities and expert bodies have established vitamin D recommendations that indicate a minimum recommended serum 25OHD level, based on minimizing the risk of developing rickets in children, or osteomalacia in adults [3, 12]. However, there is a lack of consensus on this minimum 25OHD threshold, with values varying from 25 up to 50 nmol/L (see Table 1). Differences between these recommended serum 25OHD thresholds could be explained by differences in the body of evidence considered, variability in the vitamin D assays [13], and the characteristics of the populations, such as calcium intake [14] and sun exposure [15].

**Table 1:** Internationally reported minimum serum 25OHD thresholds to ensure adequate bone health used to set vitamin D DRVs in children (ordered by increasing serum 25OHD threshold)

Agency	Location	Year	References	Serum 25OHD threshold
Scientific Advisory Committee on Nutrition (SACN)	United Kingdom	2016	[18]	25 nmol/L
World Health Organization (WHO)	Global	2004	[4]	27 nmol/L
Nutrient Reference Values (NRV)	Australia and New Zealand	2017	[48]	27.5 nmol/L
Institute of Medicine (IOM)	United States of America and Canada	2016	[12]	30 nmol/L
Global Consensus Recommendations on Prevention and Management of Nutritional Rickets	Global	2011	[19]	30 nmol/L
Indian Academy of Pediatrics (IAP)	India	2017	[49]	30 nmol/L
Nordic Nutrient Recommendations (NNR)	Nordic countries	2012	[50]	50 nmol/L
European Food Safety Authority (EFSA)	Europe	2016	[3]	50 nmol/L

Note: To convert 25OHD values from nmol/L to ng/mL, multiply by 0.40

The present systematic review and individual participant data (IPD) meta-analysis was commissioned by the FAO-WHO with the key objective of determining a serum 25OHD threshold, based on the risk of rickets, to inform the setting of the vitamin D DRV for young children. In particular, emphasis was placed on the determination of a serum 25OHD threshold in the setting of adequate dietary calcium intake. This is important because of the DRV convention that setting a vitamin D intake requirement is based on the assumption that the intake of calcium and all other nutrients is adequate [3, 12, 16]. Of note, other authorities and expert bodies thus far were unable to include this aspect in their consideration of serum 25OHD thresholds.

## Methods

The present systematic review and meta-analysis, including IPD analyses, follows the guidance provided as part of the Preferred Reporting Items for Systematic Reviews and Meta-Analyses (PRISMA)-IPD statement [17]. Approval by a research ethics committee to conduct the IPD meta-analysis was not required because the aim of this secondary analysis was consistent with the ethical approval received for the individual studies. The current analysis was conducted on anonymized data.

### Eligibility criteria

Studies involving generally healthy (apart from rickets) children below 4 years of age with total serum 25OHD levels (referred to as 25OHD henceforth) and radiologically confirmed active rickets were included. Studies in which the presence or absence of rickets were diagnosed only clinically or biochemically, but not radiologically, were excluded (to lower risk of misdiagnosis). Children 4 years and above or with conditions, such as low birth weight, prematurity, hereditary rickets, vitamin D resistant or dependent rickets, were excluded. If only serum 25OHD_2_ or 25OHD_3_ was measured, the study was excluded. The following study designs were included: cross-sectional, cohort, case–control, case report, case series, surveillance studies, before-after studies, and trials. Conference abstracts, systematic reviews, commentaries, and editorials were excluded. There were no restrictions related to the geographical location or the language.

### Search strategy

A systematic search of Embase was conducted on 7 June 2022. The search strategy is shown in Appendix 1. The search was supplemented with a manual screening of the reference lists of included articles, reviews and key international vitamin D DRV reports from other authoritative bodies [3, 12, 18–21]. Study selection was conducted in duplicate by two reviewers.

### Data collection processes, data items, IPD and data protection

Information on the characteristics of the study and their participants, the 25OHD measurement methods, as well as the method of estimation of calcium intake were extracted by one reviewer and verified by a second reviewer. Aggregate- and individual-level data (where available) for serum 25OHD and calcium intake were extracted. For before-after studies and trials with vitamin D supplementation, only the baseline data were extracted.

In the case of those identified priority studies that reported having measured calcium intake as well as serum 25OHD, collaboration, in the form of IPD sharing, was requested. The authors of each study were contacted by e-mail (up to a maximum of 3 times). For willing collaborators, data were initially de-identified at source before encryption and transfer by e-mail. In line with recently published principles and recommendations in relation to the sharing and reuse of IPD [22], data within the individual data files were used to establish an overall anonymized data file, as follows: only data on the prioritized IPD variables within the transferred files were included, there were no personal identifiers included. The anonymized data file was held in Excel® V15.30 (Microsoft Corporation, USA).

### Data analysis

The statistical analyses were conducted in the graphical user interface RAnalyticFlow (version 3.1.8) with R (version 3.6.3). Serum 25OHD values were transformed into the common unit of nmol/L and calcium intake into mg/d, using the conversion factors 2.496 mol/g for 25OHD and 24.95 mmol/g for calcium. If means and standard deviations were not reported, they were estimated using medians, interquartile ranges, confidence intervals, standard errors, *t* values, *P* values, *F* values [23]. If data was only available in plots, it was extracted using PlotDigitizer [24]. Non-detectable levels of serum 25OHD were imputed using the midpoint between the detection level of the assay and zero.

The data distributions of the study-level estimates and individual-level data were plotted in histograms and outliers reviewed. Data were subjected to random effects multi-level meta-analyses, and ninety-five percent confidence intervals (95% CI) were computed. Studies that could not be meta-analyzed were summarized in a narrative manner.

The odds of having rickets at different serum 25OHD thresholds were calculated. The sensitivity (i.e. percentage of the population with disease correctly identified by the threshold) and specificity (i.e. percentage of the population without the disease correctly identified by the threshold) of different serum 25OHD thresholds to detect rickets were calculated and plotted as a receiver operating characteristic (ROC) curve. The maximal Youden index was calculated and used to determine at which serum threshold the sensitivity and specificity were maximized and thus represents the maximum potential effectiveness of a biomarker like serum 25OHD.

The sensitivity and specificity analyses were performed on the IPD subset of individuals with adequate calcium intake (as newly defined by FAO-WHO, i.e., Average Nutrient Requirement (ANR) values for 0–6 months-old, 210 mg/d, 7–11 months-old, 330 mg/d, and 1–3 year-olds, 490 mg/d) (Personal communication from Dr Jason Montez, WHO Scientist) as well as on the entire IPD dataset (irrespective of dietary calcium intake). To assess the robustness of the results, further sensitivity analyses were conducted. One sensitivity analysis was done including only IPD data with known adequate calcium intakes assessed by multiple 24h recalls. To be able to include IPD data where calcium intake was not reported, an additional sensitivity analysis was conducted with imputed missing calcium intake data. Where calcium intake was missing, it was assumed to be adequate in infants exclusively breastfed and in children with a diversified diet, including dairy products, and assumed to be insufficient in infants below 6 months of age with mixed feeding and in children above 5 months exclusively breastfed, with a low and null dairy intake, special unbalanced or vegan diet.

## Results

### Study characteristics

**Fig. 1:**
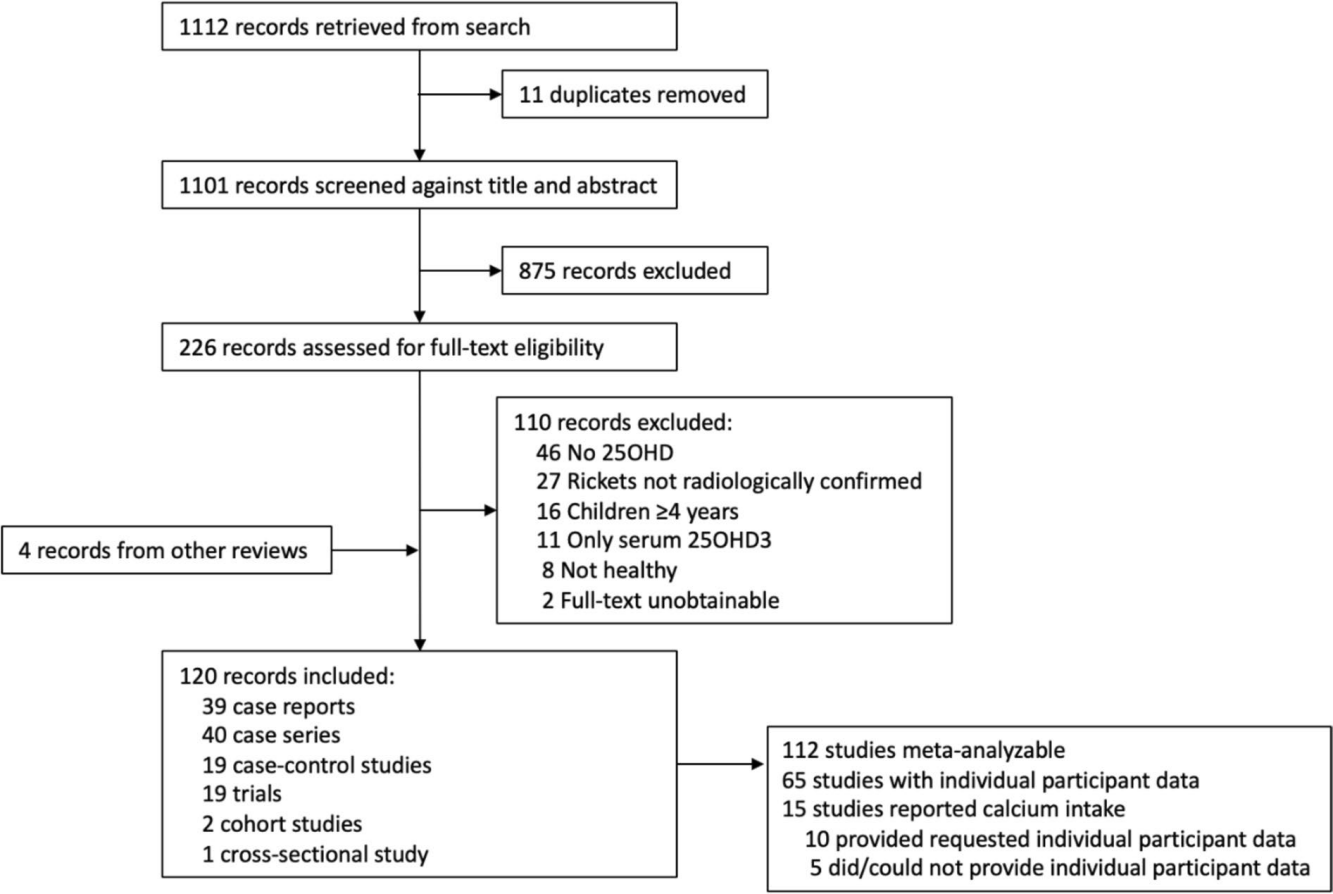
Flow chart for study selection

From a total of 1112 records identified within the search, a total of 120 studies with 5412 participants (mean age 17 months) were included (see Fig. 1). The majority of the studies were case reports (*N* = 39) and case series (*N* = 40), followed by case–control studies (*N* = 19) and trials (*N* = 19), cohort studies (*N* = 2) and a cross-sectional study (*N* = 1). The studies were conducted in all regions of the world, except Latin America. The countries in which most of the studies were conducted were the United States of America (*N* = 22), Nigeria (*N* = 14), India (*N* = 12), and Turkey (*N* = 11). The studies covered latitudes from 60.5°N to 40.9°S (mean 31.4° N). In the majority of the studies (79%), the skin pigmentation of the participants was dark. While most of the studies did not report which method was used to measure circulating 25OHD (*N* = 63), the remainder reported the use of competitive binding radioimmunoassay (*N* = 44), chemiluminescence immunoassay (*N* = 8), liquid chromatography-tandem mass spectrometry (LC–MS/MS) (*N* = 4), or high-performance liquid chromatography (HPLC) (*N* = 1). Only two studies reported participating in a vitamin D assay standardization program. The characteristics of the included studies are shown in Table 2.

**Table 2:** Study characteristics of studies of rickets in young children

Study ID*	Country	Study design	Participant description	Vitamin D assay
Acoglu 2020 [40]	Turkey	Cross-sectional study	77 Syrian and Iraqi refugee children, 1–24 months old (mean 11 months), who presented at the hospital for various reasons. Rickets definition: Inadequate vitamin D (< 12 ng/mL) and/or calcium with elevated ALP and PTH and radiological findings of rickets.	NR
**Aggarwal 2012** [35]	India	Case–control study	135 children, 0.5–5 years old (mean 13 months), with nutritional rickets (*n* = 67) and age- and sex-matched healthy controls (*n* = 68), 40% breastfed. Excluded children with non-nutritional rickets, hypocalcemic seizures, consuming calcium or vitamin D supplements in the past 6 months.	Electrochemiluminescence immunoassay (Cobas)
**Aggarwal 2013** [36]	India	Trial	67 children, 0.5–5 years old (mean 18 months), with nutritional rickets. Excluded children with features suggestive of non-nutritional etiology, hypocalcemic seizures, or consumption of calcium or vitamin D supplements in the past 6 months. This study did not report serum 25OHD at baseline.	Electrochemiluminescence immunoassay (Cobas)
**Ahmed 2020** [37]	Bangladesh	Case–control study	48 children, 1.0–10.9 years old (mean 3 years), with active rickets (*n* = 24) and their controls (*n* = 24) matched for age, sex and village. Excluded those who took medication or dietary supplements that could affect bone metabolism, with renal or intestinal disease, physical disability or impaired mobility.	Chemiluminescence immunoassay (Liaison, Diasorin), participating in the Vitamin D Standardization Program (VDSP)
Al-Atawi 2009 [51]	Saudi Arabia	Case series	283 Saudi infants, 6–14 months old (mean 9 months), with nutritional rickets, 70% exclusively breastfed with no supplementation, seen at the hospital over a 10-year period. Excluded children with liver disease, renal disease, hypoparathyroidism, taking anti-convulsion medication, and with non-nutritional rickets.	NR
Alouf 2005 [52]	USA	Case report	One female, 3.5 months old, born at term, African-American.	NR
Amirlak 2008 [53]	United Arab Emirates	Case report	Two infants, 9 months old, exclusively breastfed, with rickets.	NR
Arnaud 1976 [54]	Canada and USA	Case–control study	7 infants 2–42 months old (mean 1.7 years old) with nutritional deficiency rickets and 9 control children (mean 2.7 years old) followed in the outpatient clinics for well-baby care or problems unrelated to skeletal disease.	Radioimmunoassay
Ashraf 2002 [55]	UK	Case series	3 children, 8, 9 and 15 months old, with (*n* = 2) and without (*n* = 1) radiological features of rickets, South East Asian ethnicity.	NR
**Balasubramanian 2003** [38]	India	Case–control study	58 children, 0.5–10 years (mean 29 months), with rickets (*n* = 24) and controls (*n* = 34) attending the pediatrics department for acute illnesses (e.g. upper respiratory tract infection).	Radioimmunoassay (DiaSorin)
Balkan 2005 [56]	Turkey	Case report	One male, 6 months old, exclusively breastfed, no vitamin D supplementation.	NR
**Baroncelli 2008** [25]	Egypt and Turkey	Case–control study	148 children, 0.5–4 years old, from hospitals with rickets (*n* = 98) and non-rachitic controls (siblings or children with minor illnesses such as upper respiratory infections) (*n* = 50), 10–70% breastfed. Excluded prematurity, renal, liver, intestinal, cardiac or central nervous system disease, chronic diseases, tuberculosis, and hereditary forms of rickets.	Competitive binding RIA (DiaSorin)
Beck-Nielsen 2009 [57]	Denmark	Case series	41 children, 4–19 months (median 11–14 months), with rickets, with (*n* = 15) and without (*n* = 26) seizures, approximately 50% Ethnic Danish, 49% breastfed or weaned within 6 months before diagnosis of rickets, 24% on milk-free diet. Excluded children with serum 25OHD ≥ 50 nmol/L.	NR
Bereket 2010 [58]	Turkey	Case series	22 children, 0.2–3 years (mean 1.3 years), with rickets. Excluded prematurity, renal, liver or intestinal disease.	Chemiluminescence immunoassay (Nichols Advantage competitive binding assay)
Betend 1981 [59]	France	Case report	One male, 4 months old, term, healthy, fed with evaporated cow milk, no vitamin D supplement.	NR
Bhimma 1995 [60]	South Africa	Case series	7 children, 1–3 years old (mean 2 years), dark skin, with no vitamin D or calcium supplementation, with vitamin D deficiency rickets (*n* = 3), with calcium deficiency rickets (*n* = 3), and with healing or healed rickets (*n* = 1). Excluded gastro-intestinal, hepatic and renal glomerular causes of rickets.	Competitive binding assay
Blok 2000 [61]	New Zealand	Case series	18 children, 3–26 months old (median 12 months, mean 14 months), with vitamin D deficient rickets (serum 25OHD < 10 µg/L and radiological evidence of rickets), 66% of Indian ethnic origin.	NR
Blond 1997 [62]	France	Case report	One infant, 3 days old, dark skin.	NR
Bloom 2004 [63]	USA	Case report	One female and one male, 15 and 16 months old, with dark skin, no dairy intake, with rickets.	NR
Brinsmead 2011 [64]	Australia	Case report	One female, 12 months old, Indian ethnicity, was born at term.	NR
Chatterjee 2017 [65]	India	Case series	191 children, 0.5–9 years, with nutritional rickets. Excluded prematurity, renal or hepatic disease, intestinal malabsorption, tumor, chronic diseases, tuberculosis, diseases of the skeletal system, hypophosphatemic rickets, phosphaturia, and vitamin D-dependent rickets types 1 and 2.	Chemiluminescence micropartical immunoassay (CMIA)
Chehade 2011 [66]	Switzerland	Case report	One infant, 16 months old, dark skin, breastfed, without vitamin D supplementation, little dairy intake.	NR
Chuang 2018 [67]	Taiwan	Case series	8 children, 12–25 months (mean 20 months), with nutritional rickets, 80% with a special diet. Excluded those with rickets of causes other than nutritional, major systematic disease or taking anti-convulsant therapy.	Radioimmunoassay (Diasorin)
Curtis 1983 [68]	Canada	Case report	3 children, 1.5, 2.75 and 3.5 years old, dark skin, breastfed, vegetarian diet, no vitamin supplements.	NR
**Dabas 2022** [26]	India	Trial	132 children, 0.5–12 years, with nutritional rickets. Excluded malabsorption, chronic kidney or hepatic disease, systemic illness or vitamin D or calcium supplement in the past 6 months.	Electrochemiluminescence immunoassay autoanalyzer (Roche Cobas e411)
Dawodu 2006 [69]	United Arab Emirates	Case series	40 children, 2–30 months (median 14 months), Arab origin, with vitamin D deficiency rickets. Excluded rickets due to malabsorption, renal or liver diseases, inherited disorders such as vitamin D-dependent rickets.	NR
DeLucia 2003 [70]	USA	Case series	143 children, 4–38 months old (mean 20.2 months), with nutritional rickets, 79% African-American, 96% history of breastfeeding, and 15% vitamin D supplementation. Three cases, 24, 24 and 9 months old, dark skin, and no vitamin D supplementation, were also presented in detail. Excluded nutritional deficiency secondary to another disease and rickets secondary to genetic or other non-nutritional etiologies.	Competitive binding assay
Duplechin 1999 [71]	USA	Case report	One female, 17 months old, dark skin, breastfed, described as “picky eater”.	NR
**El Kholy 2017** [27]	Egypt	Case–control study	109 rachitic children and 30 controls (siblings or children with minor illnesses), 7 months—3 years (mean 18 months). Excluded vitamin D or calcium supplementation prior to 6 months, kidney disease, tuberculosis, liver disease, prematurity, intestinal, cardiac, central nervous system or other chronic disease, other bone disease, hereditary rickets, or low weight for height.	Immunoassay (Immunodiagnostik)
Elidrissy 1984 [72]	Saudi Arabia	Case–control study	51 children, 4–26 months old (mean 10.5 months), 92% breastfed, with active rickets (n = 16), healing rickets (n = 18) and no rickets (n = 17).	Competitive binding assay
Elidrissy 2012 [73]	Saudi Arabia	Case series	136 children with active rickets (n = 86, mean age 17 months) and healed rickets (n = 50, mean age 23.9 months).	NR
Elzouki 1989 [74]	Libya	Case series	16 children, 3–24 months (mean 15 months), treated for rickets at the hospital. Majority of the children were breastfed, infrequently exposed to sunshine, veiled mothers, malnourished. None had malabsorption.	UV absorptiometry and competitive protein binding assay
Eren 2015 [75]	Turkey	Case report	One male, 3 months old.	NR
Estrade 2017 [76]	France	Case report	One male, 2 years old, no vitamin D supplementation and his older brother, 3.2 years, and younger sister, 0.7 years, dark skin.	NR
Eugster 1996 [77]	USA	Case series	7 children, 6–20 months old (mean 10 months), 57% dark skin, with (*n* = 6) and without (*n* = 1) radiologically confirmed rickets.	NR
Fidan 2017 [78]	Turkey	Case series	26 children, 3–30 months old (mean 8.6 months), well-nourished	NR
Flot 2020 [79]	France	Case series	38 children, 0.3–12.0 years (median 1.9 years), 78% exclusively breastfed, with (*n* = 8) and without (*n* = 30) seizures, 50% Sub-Saharan African, 34% North Africa and Middle East, 11% Europe, 5% South Asia.	NR
Gad 2014 [80]	UK	Case report	One female, 7 months old, dark skin, seizures, exclusively breastfed, no supplementation.	NR
Garabedian 1983 [81]	Belgium and France	Case series	20 children, 4 months—12 years old (75% 4–26 months old), 80% immigrants, no anticonvulsant therapy.	Competitive binding assay
Ginat-Israeli 2003 [82]	Israel	Case report	One male, 2 years old, ate only a peanut snack and artificial raspberry juice, and female, 7 months old, fed diluted cow milk. Both are from Ethiopia.	NR
**Graff 2004** [28]	Nigeria	Case–control study	15 children, 2–8 years old (mean 46 months), with rickets and age- and 15 sex-matched control children (mean 47 months old). Excluded renal or liver disease, tuberculosis, chronic diarrhea, use of phenytoin, calcium or vitamin D supplements in the past 4 weeks.	Competitive binding assay
Hoecker 2002 [83]	USA	Case report	One male, 13 months old, dark skin, breastfed, no intake of soy, dairy or eggs.	NR
Holick 2009 [84]	USA	Case report	One male, 9 months old, African American, breastfed, no vitamin supplementation.	NR
Jain 2011 [41]	India	Cohort study	98 infants, 2.5–3.5 months old (mean 3.1 months), term, AGA, 71% exclusively breastfed.	Radioimmunoassay (DiaSorin)
**Jones 2018** [29]	Kenya	Case–control study	21 children, 3–24 months old (mean 12 months old), with rickets, and 22 controls without rickets or acute malnutrition. Excluded emergency medical care, tuberculosis, facture in past 3 months, HIV infection or exposure.	Radioimmunoassay (DiaSorin)
Khan 2020 [85]	Pakistan	Trial	198 children, 0.5–3 years (mean 13 months), with rickets. Excluded non-nutritional rickets, congenital anomalies, kidney or liver disease, malabsorption, antiepileptics, and history of vitamin D megadose.	NR
Koseick 2007 [86]	Turkey	Case report	One male, 14 months old, breastfed, no supplementation.	NR
Kreiter 2000 [87]	USA	Case series	23 children, 5–24 months (mean 14 months), African American, breastfed.	NR
Kruse 2000 [88]	Germany	Case series	115 children, 1 month—4.2 years old (median 13 months), with vitamin D deficiency rickets, 63% immigrants.	NR
Kubota 2006 [89]	Japan	Case series	One female, 19 months old, and one male, 29 months old, with rickets, both breastfed, male no diary intake.	NR
Ladhani 2004 [90]	UK	Case series	65 children, 0–13 years old (mean 2.6 years), with vitamin D deficiency rickets (25OHD < 25 nmol/L and radiologically confirmed rickets), 60% Asian, 37% Afro-Caribbean, 3% European, with (*n* = 29) or without (*n* = 36) hypocalcemic symptoms.	Radioimmunoassay (ImmunoDiagnostic)
Lautatzis 2019 [91]	Canada	Case series	114 children, 0–16 years (median 30 months), with nutritional rickets (*n* = 46) or vitamin D deficiency (*n* = 68). Children with 25OHD < 30 nmol/L had radiography.	LC–MS/MS or radioimmunoassay (Diasorin)
Lazol 2008 [42]	USA	Case series	58 children, 2–132 months old (mean 18 months), with nutritional rickets, 96% born full-term, 81% African Americans, 14% Arabic, 3% Hispanic, 2% Caucasian, 96% breastfed, no vitamin supplements.	NR
Lemoine 2020 [92]	France	Case report	One infant, 13 months old, Eurasian, breastfed, vegan diet, no vitamin D supplementation.	NR
Lin 2020 [93]	USA	Case report	One female, 6 months old, formula-fed.	NR
Machiels 1995 [94]	Belgium	Case report	One female, 13 months old, no dairy intake.	NR
Markestad 1984 [95]	Norway	Cohort study	7 infants followed at 3, 5, and 12 months of age.	Radioimmunoassay
Meyer 2017 [96]	Norway	Case series	37 children, 0.1–3.5 years (mean 1.4 years), with nutritional rickets (25OHD < 12.5 nmol/L or 25OHD 12.5–25 nmol/L and elevated alkaline phosphatase or PTH or low serum calcium or 25OHD 25–37 nmol/L with rickets in x-ray), 93% non-Western immigrant background.	NR
Mittal 2014 [97]	India	Trial	76 children, 0.5–5 years old (median 12 months), with rickets. Excluded malabsorption, liver or renal insufficiency, hypercalcemia, history of vitamin D, calcium or medication affecting vitamin D metabolism in the past 6 months.	Radioimmunoassay (DiaSorin)
Mittal 2019 [98]	India	Trial	86 children, 0.5–5 years old (median 10.5 months), with rickets. Excluded malabsorption, steroids, antitubercular or antiepileptic drugs, history of vitamin D, calcium or medication affecting vitamin D metabolism in the past 6 months or non-nutritional rickets.	Radioimmunoassay (DiaSorin), participating in the Vitamin D External Quality Assessment Scheme (DEQAS)
Molla 2000 [99]	Kuwait	Case–control study	103 children with rickets (mean age 14 ± SD 5 months) and 102 age- and socioethnic-matched controls (mean age 15 ± SD 6 months).	Radioimmunoassay
Moncrieff 1974 [100]	UK	Case report	One female, 4 days old, born at term, Asian ethnicity.	NR
Mondal 2014 [101]	India	Trial	61 children, 0.5–5 years old (mean 13 months), with rickets. Excluded non-nutritional rickets, vitamin D or calcium in the past 6 months.	Radioimmunoassay (DiaSorin)
Mughal 1999 [102]	UK	Case report	6 children, 10–28 months old (mean 18 months), with rickets, breastfed exclusively or prolonged periods of time, no vitamin D supplementation, dark-intermediate skin.	NR
Mustafa 1999 [103]	Canada	Case report	One male, 5 months old, breastfed, no supplement, dark skin.	NR
Naik 2017 [45]	India	Trial	110 children, 6 months old, whose mother received (*n* = 53) or not (*n* = 57) vitamin D supplementation during post-partum, breastfed.	Radioimmunoassay (DiaSorin)
Oginni 1996 [104]	Nigeria	Case–control study	26 children, 1–5 years old, with rickets and healthy controls.	Radioimmunoassay (ImmunoDiagnostic)
Oginni 2003 [105]	Nigeria	Case series	26 children, 2–5 years (mean 3.1 years), with rickets.	Radioimmunoassay (Incstar)
Ojeda 2010 [106]	Spain	Case report	One female, 5 months old, breastfed, dark skin.	NR
Olgun 2003 [107]	Turkey	Case report	One female, 9 months old.	NR
**Oramasionwu 2008** [30]	Nigeria	Case series	12 children, 2–14 years old (mean 38 months).	NR
Orbak 2005 [108]	Turkey	Case series	42 infants, 1–3 months (mean 2 months), 83% exclusively breastfed.	Radioimmunoassay (Biosource)
Ozkan 2009a [109]	Turkey	Case–control study	39 children, 0–36 months old (mean 10 months), with vitamin D deficiency rickets and 15 controls.	Competitive binding RIA (Immunodiagnostic Systems)
Ozkan 2009b [110]	Turkey	Trial	21 children, 2–16 months old (mean 7 months), with vitamin D deficiency rickets. Excluded children with familial rickets, kidney, liver and gastrointestinal system diseases.	Competitive binding RIA
Pearson 2010 [111]	USA	Case report	One male, 16 month-old, breastfed up to 12-month-old, Hispanic, with cow milk allergy, fed rice milk.	NR
Pedersen 2003 [112]	Denmark	Case series	31 children, 0.5–4 years (mean 1.7 years), 100% immigrants.	NR
Pedrosa 2013 [113]	Portugal	Case report	One female and three males, 4 months, 8 days, 9 months and 4 months old, breastfed, no supplements, dark skin.	NR
Perez-Rossello 2012 [114]	USA	Case–control study	36 children, 8–24 months old (mean 11.4 months), with vitamin D deficiency (25OHD ≤ 20 ng/mL) with (*n* = 2) or without (*n* = 34) rachitic changes in x-ray. Excluded chronic disease, oral glucocorticoids, anticonvulsants, or other medications affecting vitamin D metabolism in the past 3 months. Rickets definition: 25OHD ≤ 20 ng/mL and radiologically rachitic changes.	Radioimmunoassay (DiaSorin)
Pietrek 1980 [115]	Poland	Case–control study	213 infants, 2–24 months old, healthy (*n* = 90) or hospitalized (*n* = 123).	Radiocompetitive assay
Prentice 2008 [116]	Gambia	Case–control study	193 children 1.1–16.4 years (mean 43 months old), with active rickets (*n* = 13) and non-active rickets (*n* = 33) and community controls (*n* = 147).	Radioimmunoassay (DiaSorin)
Rajah 2008 [117]	United Arab Emirates	Trial	16 children, 6–48 months (mean 17 months), with nutritional rickets with vitamin D deficiency (*n* = 8) or calcium deficiency (*n* = 8). Excluded hypocalcemia-related tetany/seizures.	Chemiluminescence immunoassay (Nichols Advantage system)
Rajah 2010 [118]	United Arab Emirates	Trial	10 children, 11–39 months old (mean 21 months), with rickets. Excluded renal or liver disease, antiepileptic medication, vitamin D dependent or hypophosphatemic rickets.	HPLC
Ramavat 1999 [119]	Kuwait	Case series	14 newborns, within 24h of birth, born with rachitic rosary and with rickets radiologically confirmed by wrist x-ray	Radioimmunoassay
Robinson 2006 [120]	Australia	Case series	126 children, 0–15 years (median age 15.1 months), with rickets, 71% breastfed, 4% White, hypocalcemia (*n* = 65) or normocalcemia (*n* = 61).	Competitive binding assay
Sakamoto 2018 [121]	Japan	Case series	Two patients, 12–26 months old, with nutritional rickets.	Radioimmunoassay
Salama 2010 [122]	Egypt	Case series	32 infants, 3–18 months (mean 8 months), breastfed, not receiving supplements, with rickets.	NR
Saluja 2021 [123]	India	Trial	66 children, 9–60 months old (mean 21 months), with rickets, 95% breastfed, 51% hypocalcemia. Excluded ill, malabsorption disorders, liver or renal insufficiency, hypercalcemia, history of vitamin D, calcium supplements or drugs affecting vitamin D metabolism in past 6 months.	Radioimmunoassay (Beckman Coulter India)
Shah 1994 [43]	USA	Case series	42 children, 5–109 months old (median 16 months), with nutritional rickets, 2 received ant-convulsant therapy and 18 followed a vegan or non-dairy diet.	NR
Shah 2000 [124]	USA	Case series	9 children, 8–23 months old (mean 16 months), with nutritional rickets.	NR
Shaikh 2006 [125]	USA	Case series	5 children, 7–24 months old (mean 16 months), with vitamin D deficiency rickets, dark skin, breastfed, no supplement, described as "picky eater". Excluded prematurity, chronic renal disease, familial hypophosphatemia, hypocalcemia, congenital and genetic abnormalities.	NR
Sodri 2021 [126]	Malaysia	Case report	One female, 22 months old, little sun exposure.	NR
Soliman 2008 [127]	Qatar	Case series	46 children, up to 3 years old (mean 13 months), with nutritional rickets (i.e. low serum 25OHD, elevated ALP, normal or low Ca, normal or low PO4, high PTH, radiological confirmation of rickets). Excluded children with heritable disorders of vitamin D metabolism.	NR
Soliman 2010 [128]	Qatar	Case series	40 children, up to 3 years old (mean 16 months), with rickets. Excluded malabsorption, liver disease, renal insufficiency, malnutrition, parenteral nutrition, vitamin D deficiency secondary to congenital disorder of vitamin D metabolism.	Radioimmunoassay (Mediagnost)
Specker 1992 [47]	China	Trial	256 term infants born in Spring or Fall in a Northern or Southern city at 3–5 days of age and at 6 months of age, randomized to vitamin D supplementation (100, 200 or 400 IU/d).	Radioimmunoassay
Spence 2004 [129]	USA	Case report	One male, 9 months old, African American, breastfed, no vitamin supplementation.	NR
Stevens 2009 [130]	USA	Case report	One male, 6 months old, African American, exclusively breastfed.	NR
**Thacher 1997** [39]	Nigeria	Case–control study	37 children, 9 months—8 years (mean 3.2 years), with active rickets, only 7% were severely malnourished, no seizures, and 37 age-matched healthy controls, recovered from acute illness with normal weight. Excluded chronic diarrhea, signs of liver or renal disease or on anticonvulsant therapy.	Radioimmunoassay
Thacher 1999 [131]	Nigeria	Trial	123 children, 1–14 years old (median 46 months), with active rickets. Excluded vitamin D or calcium supplement in past 12 weeks, renal disease, tuberculosis, liver disease.	Radioimmunoassay
**Thacher 2000** [31]	Nigeria	Case–control study	246 children, 1–14 years old (median 44 months), with active rickets (*n* = 123) and controls (*n* = 123). Excluded vitamin D or calcium supplement in past 12 weeks, renal disease, tuberculosis, liver disease.	Radioimmunoassay
Thacher 2006 [132]	Nigeria	Case series	16 children, 15–48 months old (mean 31 months), with active rickets.	Radioimmunoassay (DiaSorin)
**Thacher 2009a** [32]	Nigeria	Trial	17 children, 28–118 months old (mean 44.5 months), with nutritional rickets.	LC–MS/MS (API 4000)
**Thacher 2009b **[33]	Nigeria	Case–control study	19 children, 2–10 years old, with rickets and 15 age-matched controls.	Radioimmunoassay (DiaSorin)
Thacher 2010 [133]	Nigeria	Case–control study	49 children, 15–120 months old (mean 43 months), with rickets (n = 28) or healthy controls (n = 21).	Radioimmunoassay (DiaSorin)
Thacher 2012 [134]	Nigeria	Trial	4 children (4 out of 647), 12–18 months old (mean 14.8 months), who had radiologically active rickets after 18 months intervention (calcium + vitamin A, ground fish + vitamin A or vitamin A alone).	Radioimmunoassay (DiaSorin)
Thacher 2013 [135]	USA	Case series	17 children, 5–27 months old (mean 13 months), with nutritional rickets.	NR
Thacher 2014 [34]	Nigeria	Trial	37 children 1–3 years old, with active rickets (radiographic score of at least 2.5). Note: Only the children below 4 years of age were included.	LC–MS/MS
Thacher 2015 [136]	Nigeria	Trial	88 children, 6–151 months (median 35 months), with active rickets. Excluded vitamin D or calcium supplement in the past 4 weeks.	LC–MS/MS
Train 1995 [137]	UK	Case report	One male, 5 months old, black.	NR
Trivedi 2020 [46]	India	Trial	114 children, at 6 months old, exclusively breastfed, from mothers given vitamin D3 supplements (4 × 60,000 IU) or placebo.	Chemiluminescence immunoassay (CLIA) (Access2 Beckman Coulter)
Uday 2018 [138]	UK	Case series	Three children of 5, 6, and 6 months old, exclusively breastfed, dark skin, with rickets.	NR
Valerio 2015 [139]	Portugal	Case report	One male, 28 months old, dark skin, breastfed, no dairy intake, no supplementation.	NR
Vanstone 2012 [140]	USA	Case report	One male 4 months old and a female 2 years old, both dark skin and breastfed.	NR
Vierucci 2017 [141]	Italy	Case report	One male, 10 month old, dark skin.	Radioimmunoassay (DiaSorin)
Vuletic 2016 [142]	Serbia	Case report	One male, 5 months old, not breastfed, no vitamin D supplement.	NR
Walter 2010 [143]	Spain	Case report	Two males, 6 and 7 months, of North Africa and Asia origin, breastfed.	NR
Weinstein 2003 [144]	USA	Case report	One male, 20 months old, dark skin, soy-based milk, no vitamin D supplementation.	NR
Wheeler 2015 [44]	New Zealand	Case series	58 children, 0.3–11 years old (median 1.4 years), with vitamin D deficiency rickets (25OHD < 50 nmol/L, elevated ALP and/or radiological rickets—77.4% with radiological changes), 87% born at full-term, 50% dark skin, 31% intermediate skin, and 19% fair skin, 93% history of exclusive breastfeeding, 9% vitamin D supplementation. Excluded chronic disease, fat malabsorption, liver disease, renal insufficiency, genetic forms of rickets, and parenteral nutrition.	NR
Williams 2008 [145]	USA	Case report	One female, 11 months old, African, breastfed, no intake of milk, no vitamins.	Radioimmunoassay (DiaSorin)
Yener 1995 [146]	Turkey	Case–control study	16 children, 7–24 months (mean 14.9 months), with rickets, breastfed during first months, well-nourished, but inadequate vitamin D intake (12 with infection on admission) and 15 controls, 6–24 months (mean 15.8 months), healthy (10 with infections).	Radioimmunoassay (Incstar, Stillwater)
Yu 2006 [147]	Canada	Case report	One male, 2-year-old, Inuit, no dairy, soy milk.	NR

Notes: NR: Not reported

^*^Studies in bold reported calcium intake

Individual data on serum 25OHD was reported for 65 studies with 930 participants (mean age 31 months old, range 0–47 months old), of which 75% had radiologically confirmed rickets and 25% did not have rickets. Sixteen studies reported having measured calcium intake. Upon request for IPD on serum 25OHD and calcium intake, 11 studies agreed and provided the data (666 participants, meaning that 71.6% of the participants in the IPD dataset had a corresponding calcium intake measured) [25–34]. The remaining five studies did not respond or were not able to provide the data [35–39]*.* Among the 11 studies for which data was provided, calcium intake was estimated by multiple 24h recalls in 8 studies, by a single 24 h recall in one study, by a 3-day food record in one study, and a food frequency in one study. Using the FAO-WHO’s age-specific ANR values, 23% of participants had adequate calcium intake and 77% had insufficient calcium intake.

### Study-level meta-analysis

The meta-analysis of all the studies (irrespective of study design and calcium intake) showed that children with rickets had a mean serum 25OHD of 23 nmol/L (N studies = 77, 95% CI 19–27), whereas children without rickets had a mean serum 25OHD of 62 nmol/L (N studies = 19, 95% CI 55–70). When restricting the meta-analysis to case–control studies (N studies = 16), the children with and without rickets had a mean serum 25OHD of 32 (95% CI 23–40) nmol/L and 64 nmol/L (95% CI 56–73) nmol/L, respectively. When restricting the meta-analysis to case reports, case series and trials, mean serum 25OHD in children with rickets was 17, 20, 26 nmol/L, respectively.

Eight studies were not meta-analyzable, because they did not report mean or median serum 25OHD. In one study [40], refugee children showing up at the hospital were screened for rickets. Of all the children screened, 28.5% had nutritional rickets, 40% had serum 25OHD < 30 nmol/L and 9% have serum 25OHD in the range 30–49 nmol/L. In another study [41], 47% of the infants had a serum 25OHD below 25 nmol/L and of those 72% had radiographic evidence of rickets. A case series [42] of children diagnosed with nutritional rickets found that 79% had serum 25OHD below 50 nmol/L. Another case series [43] of children diagnosed with nutritional rickets found that 62% had serum 25OHD below 25 nmol/L. A surveillance study [44] found that among children with serum 25OHD below 50 nmol/L, 77% had radiological changes associated with rickets.

Two trials [45, 46] compared 6-month-old exclusively breastfed infants of women who received vitamin D supplementation or no supplementation during postpartum. The first trial [45] found that 44% and 75% of children from non-supplemented mothers had serum 25OHD below 25 and 50 nmol/L, respectively; whereas 8% and 25% of children from vitamin D-supplemented mothers had serum 25OHD below 25 and 50 nmol/L, respectively. In the unsupplemented and supplemented groups, 3.4% and 3.6% of the children developed radiological rickets, respectively. In the second trial [46], the equivalent estimates for the development of radiological rickets in unsupplemented and supplemented children were 4% and 2%, respectively. One trial [47] found that at baseline, at 3–5 days of age, that 3% and 6% of infants with serum 25OHD < and > 27.5 nmol/L showed wrist ossification centers. At 6 months of age, after vitamin D supplementation with 100, 200 or 400 IU/d, none of the children showed any radiological signs of rickets [47].

### IPD meta-analysis

**Fig. 2:**
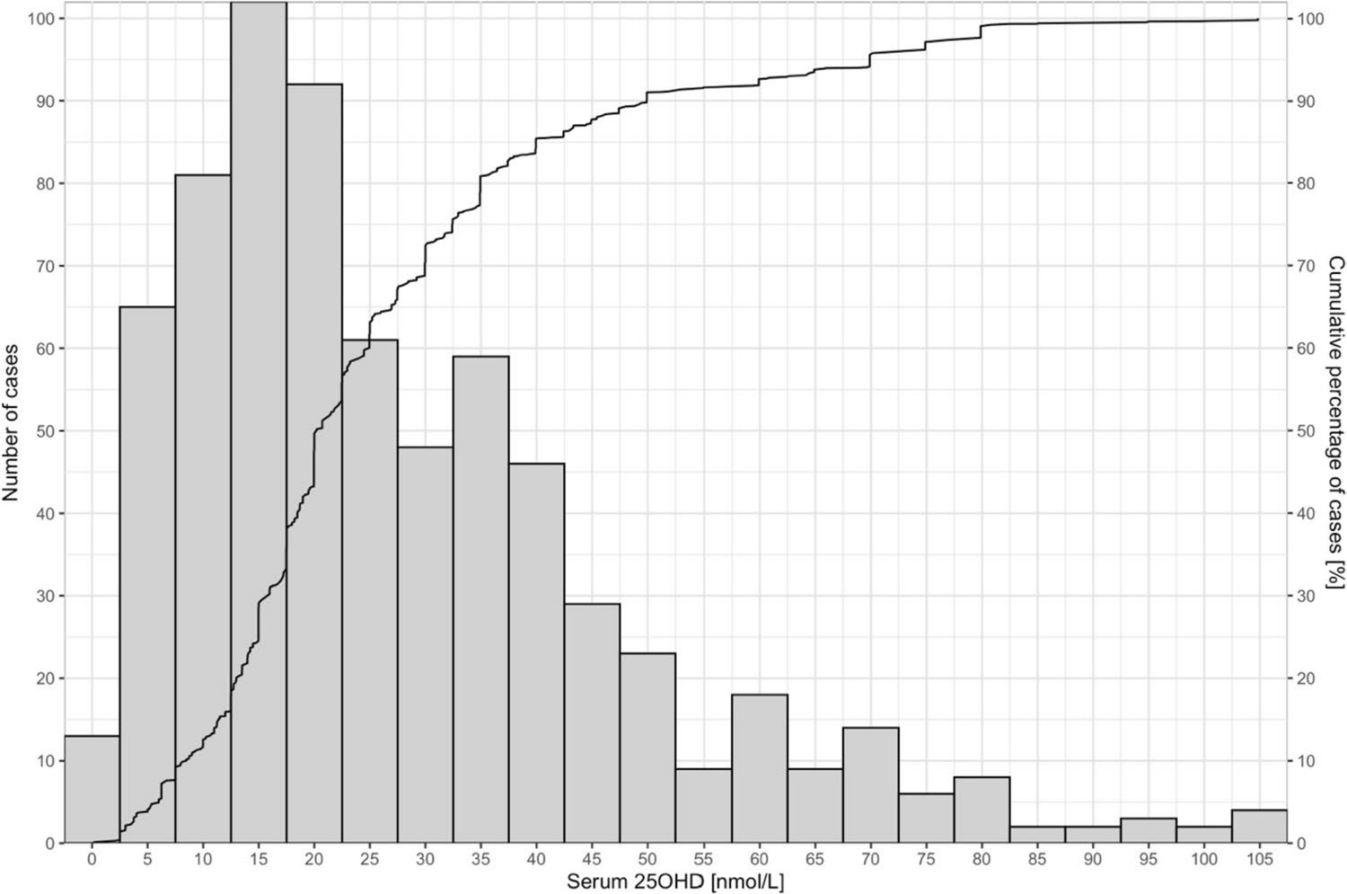
Serum 25OHD [nmol/L] distribution in children with rickets (*n* = 700)

Based on individual data and irrespective of dietary calcium intake (*n* = 930, mean 289 mg/d, median 230 mg/d), the serum 25OHD in 700 children with rickets ranged from non-detectable to 180 nmol/L, with a median of 23 nmol/L and mean of 29 nmol/L (95% CI 27–31). The distribution of the serum 25OHD in children with rickets is shown in Fig. 2. More than half (55%) of the children with rickets had serum 25OHD below 25 nmol/L, 62% below 30 nmol/L, 79% below 40 nmol/L, and 87% below 50 nmol/L. In 230 children without rickets, the median serum 25OHD was 57 nmol/L, with a mean of 62 nmol/L (95% CI 58–66).

**Fig. 3:**
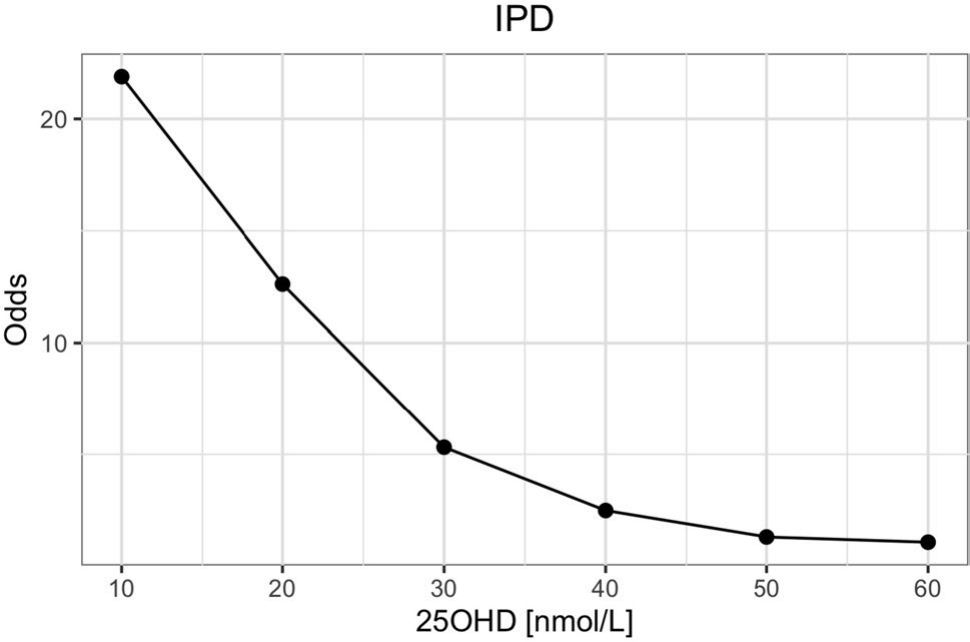
Odds of developing rickets at different serum 25OHD thresholds [nmol/L] (*n* = 930)

**Fig. 4:**
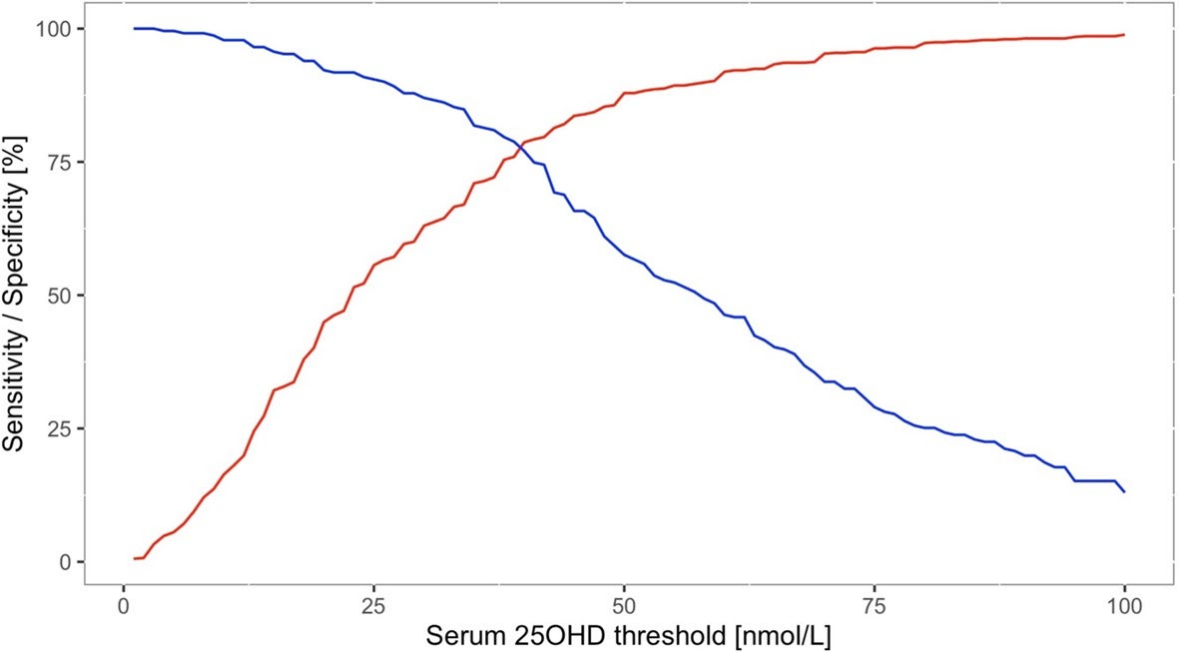
Sensitivities (red line) and specificities (blue line) for different serum 25OHD thresholds [nmol/L] (*n* = 930)

The odds of having rickets increased exponentially as serum 25(OH)D concentrations decreased below 50 nmol/L, and dramatically so when concentrations fell below 30 nmol/L (see Fig. 3). The sensitivities and specificities of different serum 25OHD thresholds to detect rickets are shown in Fig. 4 and Supplemental Table 2. A sensitivity and specificity of 80% were reached at serum 25OHD concentrations of 42 and 38 nmol/L, respectively. The serum 25OHD threshold at which the sensitivity and specificity were maximized, i.e. the maximal Youden index, was at 40 nmol/L (sensitivity 79% and specificity 77%).

#### Including only children with adequate dietary calcium intake

**Fig. 5:**
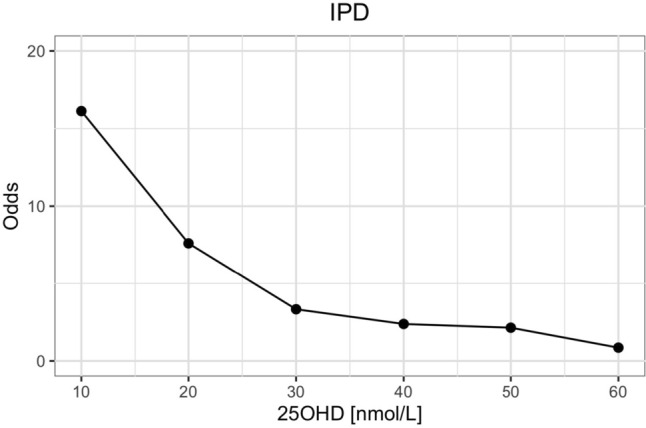
Odds of developing rickets at different serum 25OHD thresholds [nmol/L] in children with adequate calcium intake (i.e. 0–6 months old ≥ 210 mg/d, 7–11 months old ≥ 330 mg/d, 1–3 years old ≥ 490 mg/d) (*n* = 154)

**Fig. 6:**
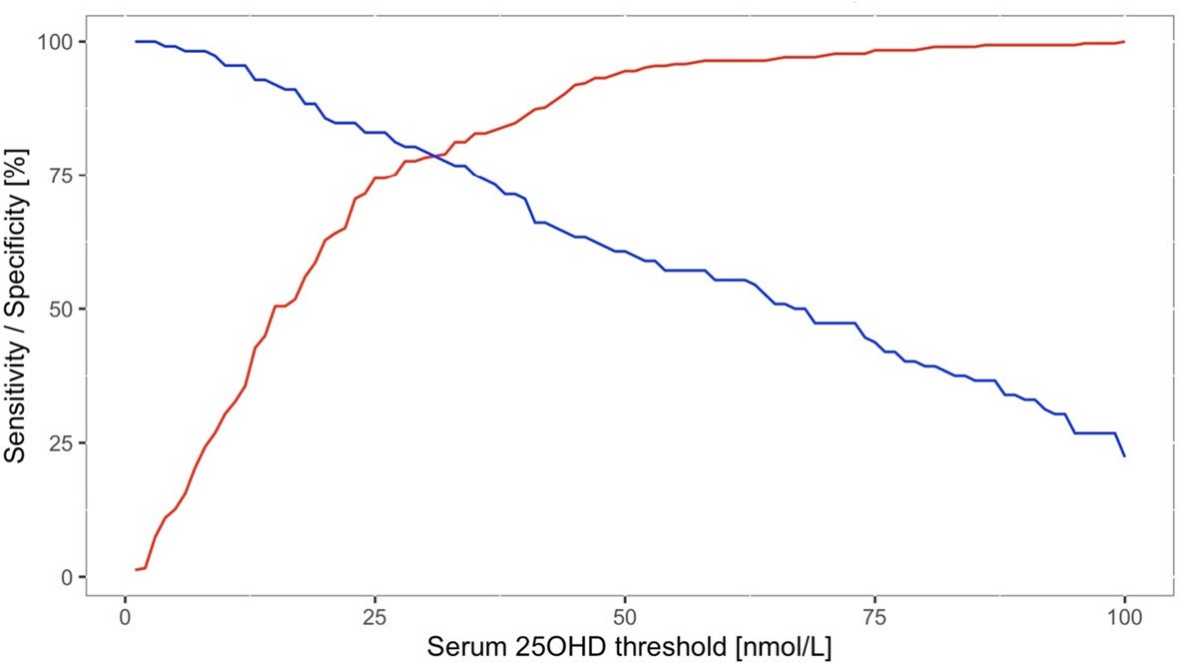
Sensitivities (red line) and specificities (blue line) for different serum 25OHD thresholds [nmol/L] in children with adequate calcium intake (i.e. 0–6 months old ≥ 210 mg/d, 7–11 months old ≥ 330 mg/d, 1–3 years old ≥ 490 mg/d) (*n* = 154)

When considering only the children with adequate calcium intakes (*n* = 640, mean 580 mg/d, median 522 mg/d), the odds of having rickets increased exponentially as serum 25(OH)D concentrations decreased below 60 nmol/L, and dramatically so when concentrations fell below ~ 25 nmol/L (see Fig. 5). The sensitivities and specificities of different serum 25OHD thresholds to detect rickets are shown in Fig. 6 and Supplemental Table 2. A sensitivity and specificity of 80% were reached at serum 25OHD concentrations of 32 and 28 nmol/L, respectively. The thresholds at which the sensitivity and specificity were maximized (i.e. Youden index) was 28 nmol/L. When including only studies with known adequate calcium intakes estimated from multiple 24 h recalls, the Youden index was at 33 nmol/L. When including studies with known adequate calcium intakes as well as assumed adequate calcium intakes (imputed), the Youden index was at 30 nmol/L.

## Discussion

In terms of the establishment of a DRV for vitamin D for young children, identification and selection of an appropriate serum 25OHD threshold is critical. This serum 25OHD concentration should protect a majority of children against increased risk of nutritional rickets and thus form a basis for derivation of a recommended dietary intake which will allow young children to maintain serum 25OHD concentrations at or above this threshold. It is not intended as a clinical threshold diagnostic for rickets.

Expert authorities charged with the establishment of vitamin D recommendations have thus far generally relied on reported individual baseline serum 25OHD concentration data in case reports, and mean/median 25OHD concentration data from studies of other designs, without trying to meta-analyze data to set a serum 25OHD threshold [3, 12, 18]. For example, the Scientific Advisory Committee on Nutrition (SACN) in the UK concluded that individual and mean serum 25OHD concentrations of children with rickets were < 25 nmol/L in the majority of studies (44 included) in their DRV exercise in 2016 [18]. The US Institute of Medicine (IOM) in 2011 identified 13 studies in their DRV exercise, and while 6 studies reported mean or median serum 25OHD concentrations < 30 nmol/L in children with rickets, the remaining studies reported mean serum 25OHD concentrations > 30 nmol/L [range 36–50 nmol/L] [12]. The European Food Safety Authority (EFSA) expert panel concluded that there is no risk of vitamin D deficiency rickets with serum 25OHD concentrations at or above 50 nmol/L and adequate calcium intake [3]. In the present extensive systematic review, the meta-analysis of study-level data also showed that young children with radiologically confirmed rickets had a mean serum 25OHD concentration of 23 nmol/L (95% CI 19–27).

A key limitation in the interpretation of such study-level data is the fact that they could be confounded by dietary calcium, especially as many of the studies were from developing countries where calcium intakes may be low [12, 18]. Thus, whether the rickets in these studies was caused solely by vitamin D deficiency and/or by low calcium intake is not clear. The present work sought to address this key knowledge gap by obtaining IPD from those studies that measured calcium intake as well as serum 25OHD in children with rickets. This data enabled an analysis of sensitivities and specificities in relation to odds of rickets at different serum 25OHD thresholds, and consequently, the estimation of the maximal Youden index, which is a measure of the potential effectiveness of a biomarker and an index used for setting optimal thresholds on medical tests. The analysis suggested the serum 25OHD threshold at which the sensitivity and specificity were maximized, i.e. the maximal Youden index, was around 28 nmol/L in children with adequate calcium, whereas this increased to 40 nmol/L in the entire sample which included children with insufficient calcium intakes. The latter would be more reflective of the types of datasets that IOM, SACN and EFSA would have based their threshold decisions upon. If dietary calcium intake is low, and serum calcium concentrations decrease, the compensatory metabolic response is an accelerated conversion of 25OHD to 1,25-dihydroxyvitamin D (via parathyroid hormone), which normalizes serum calcium concentrations [18]. This increased 25OHD catabolism leads to an increased vitamin D requirement. The IOM have suggested that when calcium intakes are inadequate, vitamin D supplementation to the point of serum 25OHD concentration up to and beyond 75 nmol/L has no effect [12]. The present findings based on empirical data from young children with adequate calcium intakes are consistent with the suggestion by the IOM, as well as other agencies briefed with the development of vitamin D DRVs, that in the face of adequate calcium intake, the risk of nutritional rickets increases below a serum 25OHD concentration of 30 nmol/L and is minimal (although not absent) when serum 25(OH)D concentrations range between 30 and 50 nmol/L [3, 12, 18]. Another report that explored the interaction between 25OHD and calcium intake from a single study, also found that the risk of rickets increased below 40 nmol/L or even higher in children with the lowest calcium intakes [14].

Two major intertwined strengths of this review are the meta-analysis of IPD, to complement the study-level meta-analyses, and that differences in calcium intake could be accounted for and the analysis be restricted to children with adequate calcium intake. The comprehensive search of the literature for the relevant studies ensured that all those studies with measured calcium intakes were identified. IPD data for 25OHD was available for 65 studies (*n* = 930) out of 120 studies (*n* = 5412). IPD was requested from the 15 studies that reported having measured calcium intakes and was obtained from 10 of these studies. The comparison of sensitivities and specificities of different thresholds allowed the identification of an optimal minimum serum 25OHD threshold. The present work also emphasized rickets as confirmed radiologically, to reduce the risk of misdiagnosing children with or without rickets.

The limitations of this review were that, due to resource and time constraints, only one online database was searched systematically. However, to ensure no important and relevant studies were missed, the reference lists of other reviews were screened. Another limitation of this review, in common with all DRV exercises to-date, is the potential variability in the serum 25OHD measurement data amongst included studies that used different analytical methods. In fact, only two of the studies included reported participating in a vitamin D assay standardization program. The measurement of serum 25OHD can vary widely between assays and participation in a vitamin D assay standardization program is recommended [13]. In addition, it was not possible to assess whether there might be different serum 25OHD thresholds on the basis of ethnicity or ancestry because the majority of the studies were of dark-skinned participants. The available data was not reported in a sufficiently consistent matter to be able to take into account sun exposure and geographical location.

In conclusion, the present IPD-level meta-analyses suggest that a minimum serum 25OHD threshold of ~ 28 nmol/L and above would represent a low risk of nutritional rickets for the majority of children with an adequate calcium intake. However, a higher 25OHD threshold is likely necessary to prevent rickets in populations with low dietary calcium intakes, which includes the geographic areas of Africa and South Asia, where rickets remain widespread. This threshold while useful within a vitamin D DRV process, as indicative of the risk of disease, it is not intended as a clinical threshold diagnostic for rickets.

### Supplementary Information

Below is the link to the electronic supplementary material.

Supplementary file1 (DOCX 16 KB)

## Data Availability

The study-level data is available upon reasonable request to the corresponding author. Individual-level data can however not be shared.
