# Supplementary material for: Serum 25-hydroxyvitamin D threshold and risk of rickets in young children: a systematic review and individual participant data meta-analysis to inform the development of dietary requirements for vitamin D
Source: Eur J Nutr. 2024 Jan 27;63(3):673–95. doi: 10.1007/s00394-023-03299-2 (PMC10948504; doi:10.1007/s00394-023-03299-2)
Supplement: Supplementary file 1 — Supplementary file1 (DOCX 16 KB) [file 394_2023_3299_MOESM1_ESM.docx]

**SUPPLEMENTARY INFORMATION**

# Serum 25-hydroxyvitamin D threshold and risk of rickets in young children: a systematic review and individual participant data meta-analysis to inform the development of dietary requirements for vitamin D

Magali RIOS-LEYVRAZ, Tom D. THACHER, Aashima DABAS, Heba Hassan ELSEDFY, Giampiero BARONCELLI, Kevin D. CASHMAN

**Supplemental Table 1. Embase search strategy**

| #1 rickets:ti,ab  #2 25$oh$d$:ti,ab OR ‘25ohd’:ti,ab OR ’25-hydrox vitamin d$’:ti,ab OR ’25-hydroxycholecalciferol’:ti,ab OR ’25-hydroxy cholecalciferol’:ti,ab OR ’25-(oh)d’:ti,ab OR ’25-hydroxyvitamin d’:ti,ab OR nutrition:ti,ab  #3 (‘animal’(exp OR ‘animal’ OR ‘nonhuman’/exp OR ‘nonhuman’ OR ‘animal cell’ OR ‘animal cell culture’/exp OR ‘animal cell culture’ OR ‘animal experiment’/exp OR ‘animal experiment’ OR ‘animal tissue’/exp OR ‘animal tissue’ OR ‘animal model’/exp OR ‘animal model’) NOT ((‘animal’(exp OR ‘animal’ OR ‘nonhuman’/exp OR ‘nonhuman’ OR ‘animal cell’ OR ‘animal cell culture’/exp OR ‘animal cell culture’ OR ‘animal experiment’/exp OR ‘animal experiment’ OR ‘animal tissue’/exp OR ‘animal tissue’ OR ‘animal model’/exp OR ‘animal model’) AND (‘human’/exp OR ‘human’))  #4 #1 AND #2  #5 rickets:ti,ab AND surve*:ti,ab AND (pediatric:ti,ab OR paediatric:ti,ab)  #6 #4 OR #5  #7 #6 NOT #3  #8 #7 NOT (‘conference abstract’/it OR ‘review’/it) |
| --- |

**Supplemental Table 2. Sensitivity and specificity of different serum 25OHD thresholds from 20 up to 50 nmol/L.**

| **Serum 25OHD**  **[nmol/L]** | **All children, irrespective of calcium intake** | | **Children with adequate calcium intake** | |
| --- | --- | --- | --- | --- |
|  | **Sensitivity [%]** | **Specificity [%]** | **Sensitivity [%]** | **Specificity [%]** |
| 20 | 45 | 92 | 63 | 86 |
| 21 | 46 | 92 | 64 | 85 |
| 22 | 47 | 92 | 65 | 85 |
| 23 | 51 | 92 | 71 | 85 |
| 24 | 52 | 91 | 72 | 83 |
| 25 | 56 | 90 | 74 | 83 |
| 26 | 57 | 90 | 74 | 83 |
| 27 | 57 | 89 | 75 | 81 |
| 28 | 60 | 88 | 78 | 80 |
| 29 | 60 | 88 | 78 | 80 |
| 30 | 63 | 87 | 78 | 79 |
| 31 | 64 | 87 | 79 | 79 |
| 32 | 64 | 86 | 79 | 78 |
| 33 | 67 | 85 | 81 | 77 |
| 34 | 67 | 85 | 81 | 77 |
| 35 | 71 | 82 | 83 | 75 |
| 36 | 71 | 81 | 83 | 74 |
| 37 | 72 | 81 | 83 | 73 |
| 38 | 75 | 80 | 84 | 71 |
| 39 | 76 | 79 | 85 | 71 |
| 40 | 79 | 77 | 86 | 71 |
| 41 | 79 | 75 | 87 | 66 |
| 42 | 80 | 74 | 88 | 66 |
| 43 | 81 | 69 | 89 | 65 |
| 44 | 82 | 69 | 90 | 64 |
| 45 | 84 | 66 | 92 | 63 |
| 46 | 84 | 66 | 92 | 63 |
| 47 | 84 | 65 | 93 | 63 |
| 48 | 85 | 61 | 93 | 62 |
| 49 | 86 | 59 | 94 | 61 |
| 50 | 88 | 58 | 94 | 61 |

Note: To convert 25OHD values from nmol/L to ng/mL, multiply by 0.40.
